# Supplementary material for: Splice-Junction-Based Mapping of Alternative Isoforms in the Human Proteome
Source: Cell Rep. Author manuscript; Available in PMC 2020 Jan 15. (PMC6961840; doi:10.1016/j.celrep.2019.11.026)
Supplement: 2 [file NIHMS1546469-supplement-2.zip › DF1/PXD006675/LeftCoronaryArtery/LeftCoronaryArtery_9_WDR73_KTTYTSGTVVLCPLLAAPAGITPQPNSATIK.pdf]

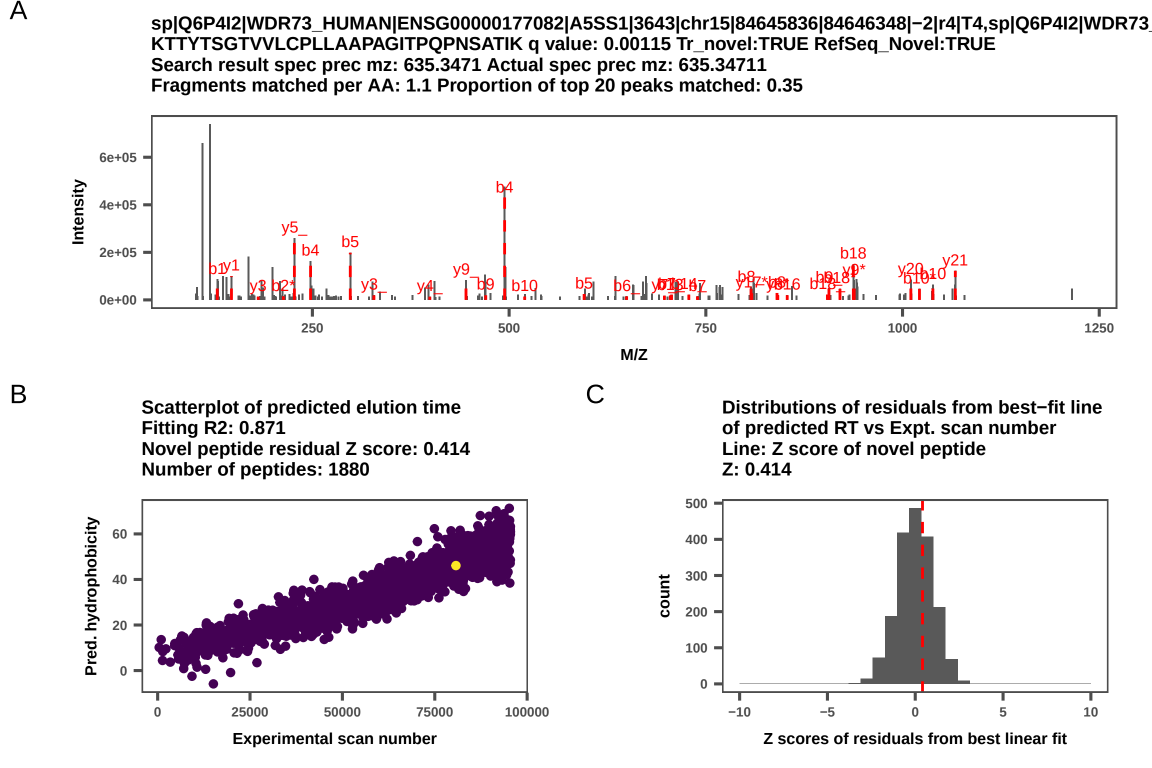
A
sp|Q6P4I2|WDR73_HUMAN|ENSG00000177082|A5SS1|3643|chr15|84645836|84646348|−2|r4|T4,sp|Q6P4I2|WDR73_
KTTYTSGTVVLCPLLAAPAGITPQPNSATIK q value: 0.00115 Tr_novel:TRUE RefSeq_Novel:TRUE
Search result spec prec mz: 635.3471 Actual spec prec mz: 635.34711
Fragments matched per AA: 1.1 Proportion of top 20 peaks matched: 0.35
Intensity
6e+05
4e+05
2e+05
0e+00
b4
y5_
b4
b5
b1
y1
y3
b2*
y3_
y4_
y9_
b9
b10
b5
b6_
b8
b18
y9*
y20
y21
b10
b16
250
500
750
1000
1250
M/Z
B
Scatterplot of predicted elution time
Fitting R2: 0.871
Novel peptide residual Z score: 0.414
Number of peptides: 1880
Pred. hydrophobicity
60
40
20
0
0
25000
50000
75000
100000
Experimental scan number
C
Distributions of residuals from best−fit line
of predicted RT vs Expt. scan number
Line: Z score of novel peptide
Z: 0.414
count
500
400
300
200
100
0
−10
−5
0
5
10
Z scores of residuals from best linear fit
